# Transmission mechanisms of an emerging insect-borne rickettsial pathogen

**DOI:** 10.1186/s13071-016-1511-8

**Published:** 2016-04-26

**Authors:** Lisa D. Brown, Kaikhushroo H. Banajee, Lane D. Foil, Kevin R. Macaluso

**Affiliations:** Department of Pathobiological Sciences, School of Veterinary Medicine, Louisiana State University, Skip Bertman Drive, SVM-3213, Baton Rouge, LA 70803 USA; Department of Entomology, Louisiana State University Agricultural Center, LSB-413, Baton Rouge, LA 70803 USA

**Keywords:** *Rickettsia felis*, Cat fleas, Transmission mechanisms

## Abstract

**Background:**

Vector-borne pathogens must overcome arthropod infection and escape barriers (e.g. midgut and salivary glands) during the extrinsic incubation period (EIP) before subsequent transmission to another host. This particular timespan is undetermined for the etiological agent of flea-borne spotted fever (*Rickettsia felis*). Artificial acquisition of *R. felis* by blood-feeding cat fleas revealed dissemination to the salivary glands after seven days; however, this length of time is inconsistent with co-feeding studies that produced infectious cat fleas within 24 h of infection. In the current study, we demonstrated that an alternative mechanism is responsible for the early-phase transmission that typifies flea-borne *R. felis* spread.

**Methods:**

Co-feeding transmission bioassays were constructed to assess temporal dynamics of *R. felis* amongst cat fleas, including exposure time to produce infectious fleas and association time to transmit infection to naïve fleas. Additional experiments examined the proportion of *R. felis*-exposed cat fleas with contaminated mouthparts, as well as the likelihood for cat fleas to release *R. felis* from their mouthparts following exposure to an infectious bloodmeal. The potential for mechanical transmission of *R. felis* by co-feeding cat fleas was further examined using fluorescent latex beads, as opposed to a live pathogen, which would not require a biological mechanism to achieve transmission.

**Results:**

Analyses revealed that *R. felis*-infected cat fleas were infectious to naïve fleas less than 24 h after exposure to the pathogen, but showed no rickettsial dissemination to the salivary glands during this early-phase transmission. Additionally, the current study revealed that *R. felis*-infected cat fleas must co-feed with naïve fleas for more than 12 h in order for early-phase transmission to occur. Further evidence supported that contaminated flea mouthparts may be the source of the bacteria transmitted early, and demonstrated that *R. felis* is released from the mouthparts during brief probing events. Moreover, the use of fluorescent latex beads supports the notion that early-phase transmission of *R. felis* is a mechanical mechanism.

**Conclusions:**

Determination of the transmission mechanisms utilized by *R. felis* is essential to fully understand the vulnerability of susceptible vertebrate hosts, including humans, to this pathogen.

## Background

*Rickettsia felis* is the causative agent of an emerging vector-borne rickettsiosis transmitted by cat fleas, *Ctenocephalides felis*, and is recognized as a common (3–15 %) cause of fever amongst hospitalized patients in sub-Saharan Africa [1–5]. In addition to the high proportion of *R. felis* infections in humans from a malaria-endemic region, the presence of this pathogen has been detected in other vertebrate hosts (including cats, dogs, opossums, raccoons, rodents and monkeys) and is present on every continent except Antarctica [6–11]. Moreover, *R. felis* has been identified in other hematophagous arthropods (including more than 40 additional species of fleas, ticks, mosquitoes, and mites) throughout the world (reviewed in [12]); nonetheless, the cosmopolitan cat flea is implicated as the primary biological vector based on field and laboratory studies [13–21]. Although maintenance of *R. felis* in nature is poorly understood, both experimental and computational transmission models indicate that this bacterium could circulate in enzootic cycles through infectious co-feeding (i.e. pathogen transmission occurs between actively blood-feeding arthropods in the absence of a disseminated vertebrate infection) by cat fleas on vertebrate hosts [22]. As such, there is a low occurrence of *R. felis* infections in the blood of vertebrate hosts and high occurrence of *R. felis*-infected arthropods in field surveys [12, 23]. Additionally, experimental demonstration of interspecific transmission of *R. felis* on a vertebrate host between cat fleas and Oriental rat fleas (*Xenopsylla cheopis*) highlights the potential for co-feeding transmission to explain the presence of *R. felis* in a variety of blood-feeding vectors [22]. Currently, the role of the vertebrate host in the transmission biology of *R. felis*, beyond providing a substrate for pathogen transfer between co-feeding arthropods, is unclear and requires further investigation.

Transmission of flea-borne bacterial pathogens is multifaceted and often each species has several transmission routes to ensure maintenance within the environment [24]. For example, agents of cat scratch disease (*Bartonella henselae*) and murine typhus (*Rickettsia typhi*) utilize horizontal transmission via contaminated flea feces deposited on the host as the primary source of infection to vertebrates [25–27]. Additional horizontal transmission occurs for these pathogens via regurgitation of bacteria from the flea’s midgut into the bite site, but requires a lengthy incubation period and occurs to a lesser extent compared to fecal transmission [24, 26]. Contrary to other flea-borne bacterial agents but similar to tick-borne rickettsial pathogens, horizontal transmission of *R. felis* can occur by infectious saliva at the bite site. Support for this saliva transmission mechanism includes identification of *R. felis* in the salivary glands of infected cat fleas [28, 29], and amplification of rickettsial DNA in the blood, as well as seroconversion, of vertebrate hosts exposed to feeding cat fleas with *R. felis* infection [14, 18]. Further evidence for transmission through infectious saliva is the transfer of bacteria between cat fleas co-feeding on a shared bloodmeal, which has been demonstrated in an artificial host system and on a vertebrate host [21, 22]. Based on the hydrodynamic force in the food canal of cat fleas (i.e. backwards, away from the bite site, whereas saliva flows forward into the bite site) and the rapid turnover of cat flea midgut contents (i.e. clearance of excessive bacteria), regurgitation of blood containing bacteria from cat fleas seems to be an unlikely scenario for transmission [30]; however, no direct evidence for or against this mechanism has been demonstrated.

The journey of an infectious agent within a vector from ingestion to subsequent transmission to a new host (i.e. extrinsic incubation period or EIP) relies on a series of complex vector-pathogen interactions [31]. Recently, the infection kinetics of bloodmeal-acquired *R. felis* in cat fleas was observed by immunofluorescence assays (IFA) at weekly intervals for 28 days [29]. This study revealed that in previously uninfected cat fleas the dissemination of *R. felis* from midgut to salivary glands requires seven or more days post-exposure (dpe) to an *R. felis*-infected bloodmeal. Based on these data, the probable EIP needed for horizontal transmission of *R. felis* by infectious cat flea saliva is approximately seven days. However, co-feeding transmission bioassays demonstrated that cat fleas exposed to an *R. felis*-infected bloodmeal are infectious to naïve fleas after 24 h (in both an artificial host system and on vertebrate hosts) [21, 22]. Thus, the EIP of *R. felis* within the biological vector remains unknown, though knowledge of this threshold is central to determining the earliest time point at which feeding *R. felis*-infected cat fleas may be infectious to a susceptible host, including humans.

Vector-borne pathogen transmission is considered biological if an incubation period is required before passage within the vector or consequent transmission to a new host [32]. In contrast, mechanical transmission does not require multiplication or development of the organism within the vector, and transmission to a new host occurs by incidental contact with the vector, such as carriage by the insects’ legs, proboscis, or gastrointestinal tract [31]. Frequently, biological and mechanical transmission of pathogens co-exists in the same geographic area, in the same hosts, and even by the same vectors [33]. Another mode of transmission has been observed for the flea-borne bacterium of plague (*Yersinia pestis*), termed “early-phase”, where transmission occurs before a designated incubation period; but, certain aspects of this transmission event have impeded confirmation as to whether this is a biological or mechanical mechanism [30]. While horizontal transmission of *R. felis* by cat fleas via infectious saliva is considered biological, the specific mechanism utilized before *R. felis* disseminates to the salivary glands is unclear. Given that *R. felis* is frequently detected in other blood-feeding arthropods, demonstration of nonspecific mechanical transfer may incriminate other human-biting vectors in the transmission cycle of this pathogen.

In this study, we aimed first to designate the EIP of *R. felis* within cat fleas, and second to further elucidate the transmission mechanism (e.g. biological or mechanical) utilized by *R. felis* amongst co-feeding fleas prior to a disseminated arthropod infection. Given that pathogen transmission before passage within the vector would indicate that microbial replication and development in the arthropod are not required, we hypothesized that a mechanical mechanism is responsible for the observed early-phase transmission of *R. felis* between co-feeding fleas. Horizontal transmission bioassays were developed in an artificial host system to assess temporal dynamics of *R. felis* between co-feeding cat fleas, including exposure time to produce infectious donor fleas and association time to transmit infection to recipient fleas. Following exposure to an infectious bloodmeal, additional experiments examined the proportion of cat fleas with *R. felis* present on their mouthparts, as well as the release of *R. felis* from food and/or salivary canals during subsequent feeding and/or probing events. The potential for mechanical transmission of *R. felis* by co-feeding cat fleas was further examined using fluorescent latex beads to simulate transfer of an inanimate object, which would not require a biological mechanism to achieve transmission. Our results indicate that not only are *R. felis*-exposed cat fleas infectious following a brief incubation period, but utilization of a mechanical mechanism may also explain the rapid rate of spread that typifies *R. felis* flea-borne transmission within experimental and computational models.

## Methods

### Source of fleas and cultivation of *Rickettsia*-infected fleas

Newly emerged, *Rickettsia*-uninfected cat fleas (*C. felis* Bouche) were purchased from Elward II (Soquel, CA, USA), and reared within an artificial host system as described previously [34]. The Louisiana State University (LSU) strain of *R. felis* was maintained in an *Ixodes scapularis* embryonic cell line (ISE6) [35], and *R. felis*-infected bloodmeals were created using an inoculation dose of 5×10^10^ rickettsiae per ml following enumeration by the *Bac*Light viability stain kit [22]. In order to differentiate between cat fleas exposed (donor) or unexposed (recipient) to a *R. felis*-infected bloodmeal, the biomarker Rhodamine B (RB) was used to label recipient fleas prior to experimentations [21].

### Experimental design

#### Kinetics of co-feeding transmission bioassays

**Fig. 1:**
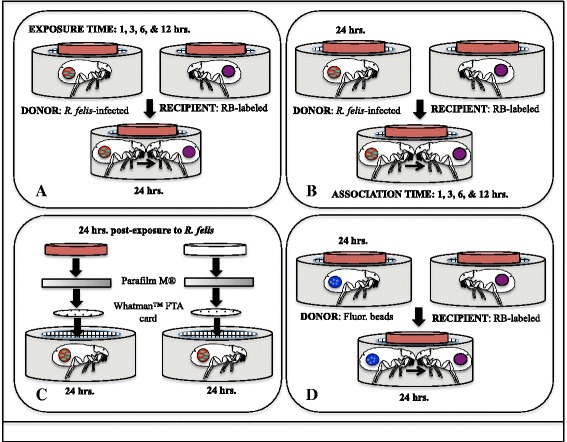
Diagrams of experimental designs. **a** Cat fleas were exposed to an infectious bloodmeal for 1, 3, 6 or 12 h, and then divided into feeding capsules containing naïve cat fleas for 24 h (exposure bioassays); **b** Cat fleas were exposed to an infectious bloodmeal for 24 h and then divided into feeding capsules containing naïve cat fleas for 1, 3, 6 or 12 h (association bioassays); **c** Whatman™ FTA cards were placed in flea cages after 24 hpe to an *R. felis*-infected bloodmeal. Cat fleas either had access to blood or the bloodmeal was removed for the duration of the experiment; **d** Cat fleas were exposed to an “infectious” bloodmeal containing fluorescent latex beads for 24 h, and then were placed with naïve fleas for 24 h

In order to examine temporal dynamics of rickettsial transmission, donor cat fleas were placed in one of two experimental groups within an artificial host system (Fig. 1a, b). The first group was exposed to an infectious bloodmeal for 1, 3, 6 or 12 h, then divided into feeding capsules containing 30 donor cat fleas and 30 recipient cat fleas for each time point (exposure bioassays, Fig. 1a). Each bioassay was conducted in three separate trials and fleas were housed together for a 24-h period on defibrinated bovine blood (non-heat inactivated). The second group was exposed to an infectious bloodmeal for 24 h, and then divided into feeding capsules containing 30 donor cat fleas and 30 recipient cat fleas (association bioassays, Fig. 1b). Each bioassay was conducted in three separate trials and fleas were allowed to co-feed together for a 1, 3, 6 or 12-h period on defibrinated bovine blood (non-heat inactivated). Immediately following each kinetics bioassay, the entire feeding capsule with all fleas was stored in the -20 °C freezer for future DNA extractions and quantitative polymerase chain reaction (qPCR) analyses. All bioassays were conducted with only female cat fleas to eliminate sexual transmission of *R. felis* within each experimental group [21].

#### Mechanism of early-phase rickettsial transmission

**Fig. 2:**
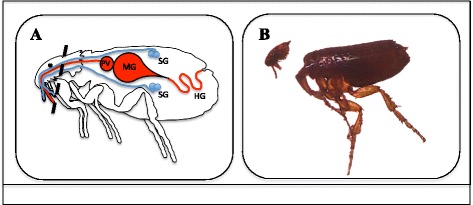
Flea dissections. **a** Diagram of flea internal anatomy. The dash line represents where the incision for dissections was made (PV,  proventriculus; MG, midgut; HG, hindgut; SG, salivary glands); **b** Photographic image of flea dissections to determine the presence of *R. felis* in flea mouthparts versus midgut at 24 hpe to an infectious bloodmeal

A two-fold approach was used to differentiate the mechanism (i.e. biological or mechanical) responsible for early-phase transmission of *R. felis* by co-feeding cat fleas. The first approach compared the presence of *R. felis* in the salivary glands *versus* the mouthparts of cat fleas following short-term exposure events. Although previous work did not detect the presence of *R. felis* in the salivary glands of cat fleas less than 7 dpe to an infectious bloodmeal [29], a portion of fleas (*n* = 100) from this study were dissected after a 24-h exposure period to confirm that original observation with a few procedural modifications (detailed below). Salivary glands from these fleas were removed, washed in phosphate-buffered saline (PBS), and then the paired tissues were either fixed with acetone onto slides for IFA (*n* = 50) or placed in microcentrifuge tubes with Buffer ATL for DNA extractions and qPCR analyses (*n* = 50). A positive control group was also dissected following the same protocol, but the salivary glands were removed from these fleas 28 dpe to an *R. felis*-infected bloodmeal. In order to determine if cat fleas harbor *R. felis* on their mouthparts in addition to their midgut at 24 h post-exposure (hpe), a portion of fleas (*n* = 70) had the upper half of their head (containing the mouthparts) removed for IFA and DNA extractions (Fig. 2). The remainder corresponding flea bodies were collected in separate tubes for DNA extraction, and flea lysates produced from both the head and body portion were analyzed for *R. felis* by qPCR. An additional group of fleas (*n* = 50) were exposed to an infectious bloodmeal for 24 h, and then permitted to feed on uninfected bovine blood for 24 h. Following this 48-h incubation period, these fleas were dissected for IFA and DNA extractions as described above. Also, Whatman™ FTA cards (filter paper designed to collect and isolate nucleic acid samples for PCR analysis; GE Healthcare™) were placed in donor flea cages after a 24-h exposure period to examine the release of *R. felis* during flea feeding and/or probing events. The Whatman™ FTA cards were placed outside the flea cages against the upper portion of the screen mesh that provides cat fleas access to blood within the artificial host system (Fig. 1c), thus ensuring that only the flea’s mouthparts had contact with the cards [36]. Two separate trials were conducted in the presence of the Whatman™ FTA cards, wherein cat fleas either had access to blood or the bloodmeal was removed for the duration of the experiment (Fig. 1c). Cat fleas were surface sterilized (10 % bleach for 5 min, 70 % ethanol for 5 min, and three rinses with sterile distilled water for 5 min each) prior to the blood-free trials in order to eliminate residual bloodmeal present on the mouthparts between feedings. For analyses, a small disc was punched from the Whatman™ FTA card, then the paper was washed per the manufacturer’s instructions (twice with FTA® Purification Reagent and twice with TE^−1^ buffer, 5 min each), and air-dried overnight before use as template for traditional PCR [35].

The second approach duplicated the co-feeding bioassays employed in a previous study [21], but instead utilized fluorescent latex beads in the place of *R. felis* infection (Fig. 1d). Product specifications for the specific beads used in this study include: (a) amine-modified polystyrene particles from Sigma-Aldrich^©^ (product number: L2778); (b) 1.0 μm mean particle size; and (c) red fluorescent dye with maximum excitation of 505 nm to 585 nm and maximum emission of 550 nm to 645 nm. Fifty cat fleas were exposed to a mock “infectious” bloodmeal containing 1×10^9^ fluorescent latex beads in 600 uL of heat-inactivated bovine blood for a 24-h period. These now “donor” fleas were then grouped together with RB-labeled recipient fleas (*n* = 50) for an additional 24 h. Following co-feeding bioassays, both donor and recipient cat fleas were dissected to remove the midgut for visual examination using a confocal fluorescent microscope (Olympus FluoView FV10i). Flea midguts were washed in PBS and placed on slides where they were mounted and counterstained using VECTASHIELD® Hard Set™ with DAPI (Vector Laboratories Inc.). Additionally, Whatman™ FTA cards were placed in donor flea cages after a 24-h exposure period to visualize the release of fluorescent beads by probing cat fleas (access to blood was not permitted). Prior to the placement of cards within flea cages as described above, cat fleas were surface sterilized to remove external beads that may have accumulated on the mouthparts. All cards were removed after 24 h and examined for beads using a fluorescent dissecting scope (Olympus MVX10).

### Detection of *Rickettsia* in fleas

For all experiments, the collected flea samples (e.g. whole fleas, individual sections, or salivary glands) were surface sterilized and genomic DNA (gDNA) was extracted using the Qiagen DNeasy Tissue Kit according to the manufacturer’s protocol and eluted in 25 μl PCR-grade H_2_O. A negative environmental control (DNA extraction reagents without biological sample) was utilized for each DNA extraction process, as well as a negative control for both PCR methods (ultrapure sterile water in the place of template). All gDNA preparations were stored at -80 °C. Quantitative and traditional PCR conditions for detection of the rickettsial 17-kDa antigen gene and the *C. felis* 18S rRNA gene were performed as described previously [17, 35]. Quantitative PCR results were presented as either quantified rickettsial copy numbers per individual flea lysate or the ratio of *R. felis* 17-kDa to *C. felis* 18S rRNA gene copy number. Amplified products from traditional PCR of Whatman™ FTA cards were visualized on 1.5 % agarose gels, and then cloned into the pCR4-TOPO vector (Invitrogen) according to the manufacturer’s protocol for DNA sequencing and analysis. At least three clones of each PCR amplicon were sequenced by the dye terminator method on a 3130 genetic analyzer (Applied Biosystems) at LSU (School of Veterinary Medicine). Sequence analyses were carried out using Vector NTI software (Invitrogen), and nucleotide similarities were compared using the GenBank database.

For the IFA, paired salivary glands were fixed in multi-well slides with ice-cold acetone for 10 min; then they were simultaneously permeabilized and blocked with 0.1 % Triton X-100 and 2 % bovine serum albumin (BSA) in PBS for 15 min. Rickettsiae were labeled with a polyclonal antibody against *Rickettsia* organisms generated in rabbits (I7198 Anti-*Rick*) and created at the National Institutes of Health’s Rocky Mountain Laboratories (generously donated by Ted Hackstadt). Anti-*Rickettsia* serum was diluted at 1:1000 in blocking buffer (0.1 % Triton X-100/2 % BSA solution), and then slides with the diluted primary antibody were incubated in the dark for 1 h. Additional slides in which no primary antibody was added served as a control for nonspecific binding, and were incubated with PBS for 1 h in the dark. Goat anti-rabbit AlexaFluor488 conjugate (Invitrogen) served as the secondary antibody, and was diluted at 1:1000 in blocking buffer (0.1 % Triton X-100/2 % BSA solution) and incubated in the dark for 1 h. Coverslips were mounted with VECTASHIELD® Hard Set™ with DAPI (Vector Laboratories Inc.) for nuclear counterstaining. Immunofluorescence assays on the upper half of the removed flea heads used an identical protocol, with the exception of the initial preparation of the tissue prior to fixation with acetone. Following flea dissections, each head was placed onto a slide within a circle drawn with a diamond point scriber (2 rows of 5 circles per slide). Multiple coverslips were placed over the entirety of the slide and the heads were then compressed between the coverslips and slide. Coverslips were then removed and discarded, and any large remnants of exoskeleton were detached from the slide with fine forceps to prevent trapping conjugate during the staining procedure [37]. All slides were visualized using a fluorescent confocal microscope (Olympus FluoView FV10i).

### Statistical analysis

A Kruskal-Wallis test was used to compare rickettsial infection loads between donor cat fleas within each kinetics bioassay, followed by a Dunn’s multiple comparison test when significance was observed. A Mann-Whitney *U*-test made comparisons within the mechanistic bioassays between total rickettsial infection loads as well as the ratio of *R. felis* to *C. felis* gene copy number between the head and body region of infected cat fleas. All statistical analyses were performed using GraphPad Prism version 6 (GraphPad Software), and differences were considered significant at *P* ≤ 0.05.

## Results

### Co-feeding transmission of *R. felis* to naïve fleas is dependent upon the exposure time to produce infectious fleas and the association time with infected fleas

In order to determine the length of time needed to produce an infectious cat flea, donor fleas were exposed to an infectious bloodmeal for 1, 3, 6 or 12 h then placed with recipient cat fleas for 24 h (exposure bioassay; Fig. 1a). In converse, donor cat fleas exposed to an infectious bloodmeal for 24 h were housed with recipient cat fleas for 1, 3, 6 or 12 h to determine the length of time needed for *R. felis* transmission to occur between co-feeding fleas (association bioassay; Fig. 1b). After 1 h and 3 h of exposure to an *R. felis*-infected bloodmeal, approximately 53 and 67 % of the donor cat fleas were positive as evidenced by qPCR, respectively; however, transmission of *R. felis* to uninfected recipient fleas was not observed at these exposure time points (Table 1). Uninfected recipient cat fleas only became positive for *R. felis* after co-feeding with infected donor cat fleas exposed for 6 and 12 h to an *R. felis*-infected bloodmeal. The 6-h exposure time point yielded an infection prevalence of 69 % in donor cat fleas and produced *R. felis* infections in 3 % of the recipient cat fleas; whereas, a 12-h exposure period resulted in an *R. felis* infection prevalence of 76 and 7 % in donor and recipient cat fleas, respectively (Table 1). Comparisons of mean rickettsial load between donor cat fleas from each exposure time point revealed no significant differences, except between the 3-h and 6-h exposure periods. Following a 24-h exposure period, infection prevalence of *R. felis* in donor cat fleas was 74, 64, 61 and 63 % in the 1-h, 3-h, 6-h and 12-h association bioassays, respectively; nevertheless, transmission of *R. felis* to uninfected recipient fleas was not observed at these association time points (Table 1). No significant difference was detected between the mean rickettsial loads of donor cat fleas from each association period. Thus, *R. felis*-infected cat fleas are subsequently infectious to others via co-feeding after a 6-h incubation period, but *R. felis* transmission to uninfected cat fleas does not occur if co-feeding with infected cat fleas is 12 h or less.

**Table 1:** Temporal dynamics of rickettsial transmission between co-feeding cat fleas. Cat fleas were either exposed to an infectious bloodmeal for 1, 3, 6 or 12 h, and then divided into feeding capsules containing naïve cat fleas for 24 h (exposure bioassay), or exposed to an infectious bloodmeal for 24 h, and then divided into feeding capsules containing naïve cat fleas for 1, 3, 6 or 12 h (association bioassay). Acquisition of novel infection by recipient fleas was assessed by qPCR. Rickettsial infection loads were determined by quantifying the copy number of a portion of the R. felis 17-kDa antigen gene per individual flea lysate

	Donor cat fleas		Recipient cat fleas	
Exposure (h)	Prevalence (%)	Mean infection load (± SEM)	Prevalence (%)	Mean infection load (± SEM)
1	48/90 (53)	4.12E^4^ (± 1.30E^4^)	0/90 (0)	0
3	60/90 (67)	3.33E^4^ (± 7.39E^3^)	0/90 (0)	0
6	62/90 (69)	4.78E^4^ (± 9.95E^3^)	3/90 (3)	2.60E^3^ (± 2.16E^3^)
12	68/90 (76)	3.27E^6^ (± 2.79E^6^)	6/90 (7)	6.12E^3^ (± 3.12E^3^)
Association (h)				
1	67/90 (74)	1.27E^7^ (± 1.12E^7^)	0/90 (0)	0
3	58/90 (64)	9.23E^3^ (± 3.12E^3^)	0/90 (0)	0
6	55/90 (61)	2.80E^4^ (± 1.35E^4^)	0/90 (0)	0
12	57/90 (63)	7.66E^4^ (± 6.24E4)	0/90 (0)	0

### Cat flea salivary glands are not the anatomical source of *R. felis* for early-phase transmission

**Fig. 3:**
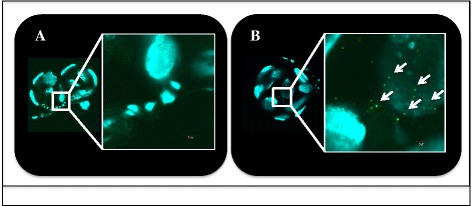
Dissemination of *Rickettsia* to flea salivary glands. **a** No rickettsial antigen is present at 1 dpe to an *R. felis*-infected bloodmeal; **b** Presence of rickettsial antigen (*labeled green, indicated by arrows*) at 28 dpe to an *R. felis*-infected bloodmeal (positive control)

In order to differentiate the mechanism (i.e. biological or mechanical) responsible for early-phase transmission of *R. felis* by co-feeding cat fleas, the presence of *R. felis* was compared between the salivary glands versus the mouthparts of cat fleas following a 24-h exposure to an infectious bloodmeal. *Rickettsia felis* was not detected in the salivary glands of cat fleas via IFA following this short-term event (24 hpe), as opposed to the positive control group where rickettsial antigen was identified 28 dpe to an *R. felis-*infected bloodmeal (Fig. 3). Quantitative PCR analyses confirmed the lack of rickettsiae at 24 h with no amplification of the *R. felis* gene in the salivary glands assessed from the same time point. Correspondingly, 10 % (7/70) of the heads removed from cat fleas were positive for *R. felis* as evidenced by qPCR after 24 h exposure to an infectious bloodmeal; however, no definitive organisms were detected via IFA. Additionally, a significant difference was observed between the average (± SEM) rickettsial load detected within the head (1.5×10^3^ ± 1.3×10^3^) and body (1.3×10^5^ ± 9.0×10^4^) between corresponding flea lysates, as well as between the ratio of *R. felis* to *C. felis* genes between the head (7.2×10^-3^ ± 6.6×10^-3^) and body (2.4×10^-2^ ± 2.0×10^-2^) segments. Moreover, 4 % (2/50) of the heads removed from cat fleas 48 hpe confirmed the presence *R. felis* by qPCR analyses, but again no definitive organisms were detected via IFA. The average (± SEM) rickettsial load detected in flea heads at 48 h (7.9×10^1^ ± 1.6×10^1^) was significantly less than flea heads collected at 24 h (1.5×10^3^ ± 1.3×10^3^), thus further decreasing the likelihood for visualization by fluorescent microscopy. Consequently, these results suggested that *R. felis* resides within the mouthparts, not the salivary glands, of cat fleas following a 24-h exposure to an infectious bloodmeal.

### Cat fleas release *R. felis* from contaminated mouthparts during probing events

**Fig. 4:**
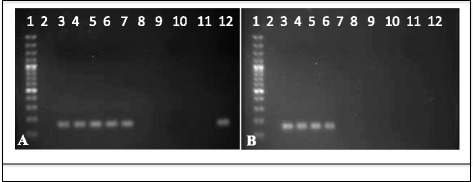
PCR detection of rickettsial 17-kDa antigen gene in Whatman™ FTA cards. **a** Lane 1, 100 bp DNA marker; Lane 2, blank; Lanes 3–7, single disc punch from five different cards exposed to *R. felis*-infected cat fleas in the presence of blood; Lanes 8–10, blank; Lane 11, environmental control; Lane 12, positive PCR *R. felis* genomic DNA; **b** Lane 1, 100 bp DNA marker; Lanes 2, 7–11, blank; Lanes 3, 4, 5, single disc punch from three different cards exposed to *R. felis*-infected, surface sterilized cats fleas in the absence of blood; Lane 6, positive PCR *R. felis* genomic DNA; Lane 12, environmental control

**Fig. 5:**
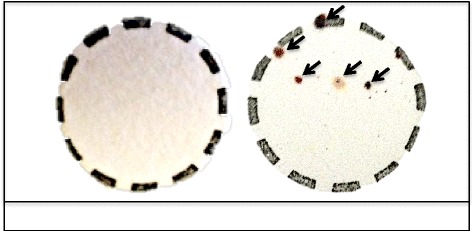
FTA cards exposed to cat fleas in the absence (*left*) and presence (*right*) of blood. Residual blood droplets (*arrows*) were deposited when cat fleas had access to blood

To determine the release of *R. felis* during cat flea feeding and/or probing events in both the presence and absence of blood, Whatman™ FTA cards were placed in donor flea cages after a 24-h exposure period. The presence of rickettsial DNA from Whatman™ FTA cards was confirmed by PCR amplification (Fig. 4a, b) and nucleotide sequences of the 17-kDa antigen (434 bp) genes were identical to those of the sequences reported for *R. felis* in the GenBank database (accession numbers CP000053 and AF195118). Interestingly, although flea mouthparts were unable to penetrate through the cards to feed in trials with access to blood (feeding occurred at the periphery not covered by paper), droplets of blood were deposited along the surface of cards exposed to these fleas (Fig. 5). In contrast, cat fleas that were surface sterilized prior to placement with Whatman™ FTA cards in the absence of blood did not leave evidence of feeding and/or probing (Fig. 5), yet *R. felis* was still detected (Fig. 4b). Thus, these data provide initial evidence for the persistence of *R. felis* within residual blood deposited from the food and/or salivary canals while probing, as well as the potential for bacteria to adhere to the inside of these stylets and consequently discharged through probing events.

### Early-phase transmission of *R. felis* is due to a mechanical mechanism

**Fig. 6:**
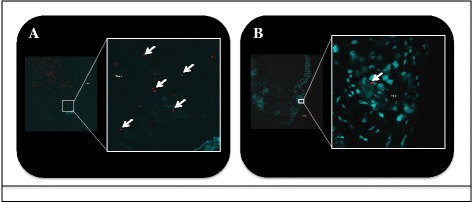
Dissections of cat flea midguts exposed to fluorescent latex beads. **a** Donor cat flea with fluorescent beads (*arrows*) after 1 day post-exposure to an “infectious” bloodmeal; **b** Recipient cat flea with fluorescent beads (*arrow*) after 1 day of co-feeding with donor cat fleas

**Fig. 7:**
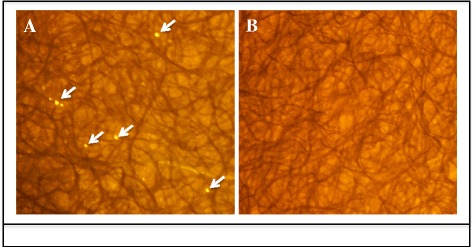
Whatman™ FTA cards placed in cat flea cages at 24 hpe to fluorescent latex beads in blood. **a** Cat fleas deposited beads (*arrows*) onto cards following surface sterilization and no access to blood; **b** Whatman™ FTA card exposed to non-experimental cat fleas with no access to blood

Given now the evidence for *R. felis* on the mouthparts of cat fleas following a 24-h exposure to an infectious bloodmeal, the potential for mechanical transmission by co-feeding fleas was further evaluated with the use of size-matched fluorescent latex beads as opposed to a live pathogen. Following a 24-h exposure to this “infectious” bloodmeal, donor cat fleas possessed large quantities of fluorescent beads within their midgut (Fig. 6a). Intriguingly, recipient cat fleas were found to harbor fluorescent beads within their midgut after co-feeding with these donor fleas for 24 h (Fig. 6b). Additionally, donor cat fleas deposited these beads onto FTA cards following surface sterilization prior to placement within flea cages with no access to blood (Fig. 7). Therefore, based on these data, the mechanism responsible for early-phase transmission of *R. felis* between co-feeding cat fleas is determined to be mechanical by this criterion.

## Discussion

In principle, there are biological, morphological, and behavioral aspects of fleas that are favorable for the transmission of any microorganism in the bloodstream of a vertebrate host; yet, there are proven significant differences between distinct vector species and their efficacy rate in transmitting a given agent of disease [38]. For example, amongst the 30 flea species confirmed as competent vectors of *Y. pestis* in North America, *X. cheopis* showed the highest proportion of pathogen acquisition (70–100 %) and transmission efficiency rates (30–70 %) [24]. However, *X. cheopis* requires a long EIP (12–16 dpe) before subsequent transmission of *Y. pestis* to others, and persistent *Y. pestis* infection is typically followed by death [24]. Although *X. cheopis* is perceived as the most efficient vector of *Y. pestis*, it transmits the plague bacterium inefficiently. Consequently, the EIP of a pathogen within a given arthropod is one of the most important factors affecting vector efficacy. The cat flea has demonstrated proficiency in both pathogen acquisition (30–100 %) and transmission efficiency rates (10–30 %) for *R. felis* in previous laboratory studies [16, 21, 22], but the length of time needed from ingestion to transmission of *R. felis* by cat fleas was not assessed. Furthermore, although *R. felis* is widely disseminated throughout the cat flea host (including the midgut epithelial cells, muscle cells, fat body, tracheal matrix, ovaries, epithelial sheath of testes and salivary glands), a correlation between rickettsial distribution in flea tissues and distinct transmission routes has not been determined [23].

Our results demonstrated that donor cat fleas are infectious as early as 6 hpe to an *R. felis*-infected bloodmeal, but do not transmit *R. felis* if the association time with recipient fleas is 12 h or less. Interestingly, although more than 50 % of the donor cat fleas were infected with *R. felis* at 1 and 3 hpe, co-feeding transmission to naïve fleas was not observed until 6 and 12 hpe in these bioassays. The initial assumption for the observed delay was that perhaps rickettsial loads within each donor flea group (1, 3, 6 and 12 hpe) influenced *R. felis* co-feeding transmission (i.e. transmission events were dose-dependent), but the only significant difference between rickettsial loads of donor cat fleas was at 3 and 6 hpe. This difference is not considered substantial because there was no transmission at 1 hpe and the highest proportion of transmission events occurred at 12 hpe; yet, rickettsial loads of donor cat fleas from 1 and 12 hpe were not significantly different from other time points. Surprisingly, co-feeding transmission of *R. felis* to recipient fleas was not observed at any association time points (1, 3, 6, and 12 h) even though more than 60 % of the donor cat fleas were infected in all groups with comparable rickettsial loads. Therefore, similar to earlier work [22], co-feeding transmission of *R. felis* between cat fleas is not dose-dependent; however, there is an incubation period required before transmission for reasons not currently understood.

Transmission of flea-borne pathogens may occur through several possible mechanisms, including: contaminated feces (e.g. *R. typhi, B. henselae*); soiled mouthparts (e.g. viral pathogens); regurgitation of gut contents (e.g. *Y. pestis*); and infectious saliva via infected salivary glands (e.g. *R. felis*) [24]. Similarities often exist between transmission routes utilized by rickettsial pathogens, but the flea-associated *Rickettsia*, *R. typhi* and *R. felis,* exhibit rather dissimilar transmission routes. Jointly, infection in the flea is initiated when ingested rickettsiae enter and replicate within the epithelial cells of the midgut. For *R. typhi*, the rickettsiae within the midgut cells are released into the gut lumen for excretion with feces at 10 dpe to an infectious host [26]. For *R. felis*, the rickettsiae migrate from the midgut cells to the salivary glands for inoculation into hosts with flea saliva, roughly a 7–14 day migration from the moment of arthropod ingestion [29]. Since the kinetics of bloodmeal-acquired *R. felis* in cat fleas was demonstrated [29], interpretation of other studies now suggests that transmission of *R. felis* by co-feeding cat fleas may occur prior to salivary gland infection [21, 22]. Similar to the kinetics account [29], the current study did not detect *R. felis* in the salivary glands of cat fleas following a short-term exposure event (24 hpe) by qPCR or IFA analyses; nevertheless, transmission of *R. felis* between co-feeding cat fleas occurs at 24 hpe or less [21, 22]. Rickettsial DNA was, however, detected by qPCR in 10 and 4 % of the dissected flea heads (encompassing the mouthparts) at 24 and 48 hpe, respectively. Although no definitive organisms were detected from the heads via IFA, this may be due to the lower sensitivity of IFA when compared to qPCR analyses. Currently, the survival of *R. felis* on the external mouthparts of cat fleas is unknown, but it is possible that bacteria present in residual blood on the posterior portion of the flea mouthparts (or anterior pharynx) could survive environmental elements from within the flea’s head capsule [30].

The dissimilar transmission routes of flea-borne rickettsial species may also reflect differences between the feeding behavior of each vector, with *C. felis* and *X. cheopis* as the recognized biological vectors for *R felis* and *R. typhi*, respectively. Because *X. cheopis* feed so infrequently, once every 1–3 days [39], there is ample opportunity for *Rickettsia* spp. to replicate and escape the midgut cells before defecation on a host. In addition to fecal transmission, further studies revealed that *X. cheopis* infected for > 21 days were capable of transmitting *R. typhi* to hosts by bite; however, oral transmission of *R. typhi* is the result of regurgitation of excess *Rickettsia* present in the gut lumen of fleas rather than through salivary secretions [40]. Due to the rapid feeding behavior (roughly 14 h total daily of intermittent feeds) and high turnover rate of gut contents [41], *R. felis*-infected cat fleas are not known to regurgitate excess bacteria from the midgut during successive bloodmeal acquisition. A more likely scenario for transmission of *R. felis* prior to salivary gland infection is that cat flea mouthparts harbor residual blood along the grooved surfaces that form the food and salivary canals [30]. The general feeding behavior of many arthropods with piercing-sucking mouthparts is performed by a series of brief probes to locate capillaries within the vertebrate [42]. During these probing events, bacteria present in the salivary grooves distal to the salivary pump would be driven into the bite site [30, 42]. Our results demonstrated that *R. felis* is released from contaminated mouthparts of cat fleas following exposure to an infectious bloodmeal as evidenced by nucleic acid isolation from Whatman™ FTA cards. Additionally, residual blood was deposited between intermittent feeds by probing cat fleas as visualized on these cards when access to blood was granted. Given that flea mouthparts were unable to penetrate through the card due to the thickness of the paper, the presence of these blood droplets is significant because it demonstrates the potential for remaining blood in the salivary canal to transfer with saliva into the next bite site. Due to the opposing hydrodynamic forces of the food and salivary canals, regurgitation of excess blood blocked before the prestomach by probing cat fleas seems unlikely [30]. Furthermore, no visual evidence of probing was demonstrated when cat fleas were surface sterilized prior to placement with Whatman™ FTA cards and given no access to blood, yet *R. felis* was still detected using the same techniques; thus, highlighting that bacteria within the salivary grooves seems most prone to transmission during probing expeditions.

In the strictest sense of the delineation between a biological and mechanical mechanism, transmission of *R. felis* by cat fleas with no discernable EIP (e.g. transfer of *R. felis* before disseminated arthropod infection) would be classified as a mechanical mechanism. Moreover, the potential for declining transmission efficiency with additional bloodmeals (e.g. the proportion and infection load of *R. felis* in the head region of cat fleas decreased between 24 and 48 hpe) indicates that the source for early *R. felis* transmission is not sufficient for multiplication and persistence of the bacteria (another qualifier for a mechanical mechanism). However, early-phase transmission of *R. felis* is not instantaneous, which is not compatible with a mechanical mechanism. A minimal incubation period is required before *R. felis* transmission may occur, but this interval is not dependent on the amount of *Rickettsia* ingested or replication of the bacteria within the flea. Similar observations have been documented for early-phase transmission of *Y. pestis*, including a short incubation period (ranging from a few hours to 1–2 dpe) and the lack of a correlation between bacterial loads and transmission events [43–50]. The combination of results from early-phase transmission experiments suggests that the location of bacteria within the flea is a more important indicator of transmission outcome than the initial amount of bacteria present [51]. Several authors [30, 52, 53] have proposed that the mechanical *vs* biological dichotomy is oversimplified, and suggested two other possible mechanisms of vector-borne transmission: ingestion-salivation and ingestion-egestion. Although currently classified as non-biological, these two mechanisms depend on adherence of the pathogen to the interior surfaces of the vector before subsequent inoculation during the next feeding event. The present study used fluorescent latex beads to demonstrate that early-phase transmission of *R. felis* by cat fleas is accomplished by a mechanical mechanism. The release of latex beads from feeding and/or probing cat fleas, as demonstrated through co-feeding bioassays and Whatman™ FTA cards, supports the notion that early-phase transmission is mechanical; however, mechanical, ingestion-salivation, and ingestion-egestion mechanisms may not be mutually exclusive. The minimal theoretical conditions required for mechanical transmission are (i) high parasitemia in donor vertebrate hosts; (ii) high density of potential mechanical arthropod vectors; (iii) high receptivity and susceptibility of a major part of potential recipient vertebrate hosts; and (iv) close contact between recipient and donor vertebrate hosts [33]. Although systemic vertebrate infections with *R. felis* remain an occasional phenomenon with highly variable frequency and impact, these minimal conditions for mechanical transmission are met when the cat flea is considered the biological vector and reservoir host for this pathogen.

Utilization of both biological and mechanical mechanisms may be extremely advantageous depending on the transmission cycle of a pathogen. The majority of our current understanding of *R. felis* transmission is derived from cat flea colonies maintained on live cats or in an artificial host system. Remarkably, exploitation of both mechanisms by *R. felis* coincides with the general ecology of cat fleas associated with domestic cats. For example, on-host longevity of cat fleas is approximately eight days due to the grooming efficiency of cats [54]. Thus, if the EIP for biological transmission of *R. felis* by cat fleas is roughly the same amount of time as the average lifespan of the vector, then a mechanical mechanism must be used to safeguard the probability of pathogen transmission. Moreover, only about 5 % of cat fleas transfer from one cat host to another every seven days [55]. An immediate transfer to a second host is favorable for mechanical transmission, but weekly transfer rates of cat fleas was previously demonstrated as sufficient for the maintenance and persistence of *R. felis* within cat flea populations [22]. Intriguingly, the current study revealed that *R. felis*-infected cat fleas must co-feed with naïve fleas for more than 12 h in order for transmission to occur. This rather lengthy association time needed to ensure *R. felis* transmission might reflect a crucial component in the vectorial capacity of cat fleas for this pathogen, such as the long-term persistent feeding behavior of cat fleas on the same vertebrate host compared to transient blood-feeding arthropods. Furthermore, migration to the salivary glands must be required for sustained transmission given that the presence of *R. felis* on the mouthparts of cat fleas declined between 24 and 48 hpe with the advent of an uninfected boodmeal. Similar results were found in a previous study when a portion of fresh blood was assessed for rickettsial DNA at 24 and 48 hpe to *R. felis*-infected cat fleas (3.3×10^3^ and 3.0×10^2^ rickettsiae per 200 μl of blood, respectively) [21]. Likely, intermittent feeding by cat fleas on the same host consists of both infected and uninfected bloodmeals because co-feeding transmission of *R felis* is dependent upon the close proximity (within a few centimeters) of infected and uninfected vectors [22]. Therefore, *R. felis* does not appear to thrive, multiply or persist in a transmissible state under a mechanical mechanism alone.

## Conclusions

The primary role of cat fleas in the transmission biology of *R. felis* has been well established; yet, transmission mechanisms utilized by *R. felis* within cat flea populations for sustaining enzootic cycles are less understood. In summary, our results demonstrate that cat fleas are infectious following a brief exposure to an *R. felis*-infected bloodmeal, and transmission of *R. felis* prior to dissemination within cat flea tissues is accomplished by a mechanical mechanism. The *R. felis*-*C. felis* relationship is truly unique in that most noncirculative, nonpersistent pathogens are generally not vector species-specific [42]; however, the demonstration of mechanical transmission may incriminate other human-biting vectors in the transmission cycle of this pathogen. A recent report has implicated mosquitoes as potential vectors of *R. felis* in regions of Africa [56], where there is currently a debate as to whether cat fleas from that area possess a unique strain of *R. felis* or a different species entirely [12]. Given that *R. felis* has been detected molecularly in numerous arthropod species across the globe, there exists the potential for geographic-dependent vectors; although, additional studies will be required to discern the biological significance of *R. felis* infection in these various arthropod hosts.

## Abbreviations

*Cf*18S: portion of *C. felis* 18S rRNA gene
DNA: deoxyribonucleic acid
Dpe: days post-exposure
EIP: extrinsic incubation period
gDNA: genomic DNA
HI: heat inactivated
hpe: Hour(s) post-exposure
hr(s): Hour(s)
IFA: indirect immunofluorescent assay
ISE6: *Ixodes scapularis* cell line
LSU: Louisiana State University
PCR: polymerase chain reaction
qPCR: quantitative real-time PCR
RB: Rhodamine B
*Rf*17kDa: portion of *R. felis* 17-kDa antigen gene
SEM: standard error of the mean
